# Genetic diversity and the ensuing strain specific immunity to asexual erythrocytic stage vaccine candidate antigens, MSP-1, AMA-1 and DBP, of *Plasmodium vivax* in Sri Lanka

**DOI:** 10.1186/1475-2875-9-S2-P55

**Published:** 2010-10-20

**Authors:** Preethi Udagama-Randeniya, Sajani Dias, Prasad Premaratne, Thilan Wickramarachchi

**Affiliations:** 1 Department of Zoology, University of Colombo, Colombo 03, Sri Lanka

## 

Characterization of genetic diversity among locally prevalent parasite strains and the ensuing strain-specific immunity will be particularly important in a specific geographical setting for development of vaccine constructs and for planning future vaccination strategies.

The present study characterized the prevailing genetic diversity of three major asexual stage putative vaccine antigens of *Plasmodium vivax,* 42 kDa fragment of the Merozoite Surface Protein -1 (MSP-1_42_), Apical Membrane Antigen-1 domain II (AMA-1 DII) and the Duffy Binding Protein region II (PvDBPII), in patients infected with *P. vivax* malaria from two endemic areas, Anuradhapura and Kataragama, in Sri Lanka, where unstable malaria prevails with low transmission, and from a malaria non-endemic area, Colombo, where the patients contracted the infections while visiting areas endemic to vivax malaria. Based on nucleotide sequences, extensive allelic polymorphism was evident in the N-terminal region of MSP-1_42_*(Pvmsp-*133*), Pvama- 1dII* and *PvdbpII,* due to recombination, mutations, natural selection and genetic differentiation. Nucleotide sequences of *Pvmsp-1_42_*(N=95), *Pvama-1dII* (64) and *PvdbpII* (100), generated 27, 21 and 33 amino acid (a.a) haplotypes, respectively. Clinical isolates with amino acid haplotypes available for all three antigens (N=24) revealed uniquely different haplotype combinations in each isolate. Conversely, PvMSP-1_19_, domain II loop of PvAMA-1 and the six a.a. in the critical binding domain of PvDBPII responsible for direct binding with the Duffy antigen on human RBC, showed 100% conservation of the amino acid sequences.

In order to evaluate whether sequence differences among each antigen was indicative of strain-specific immunity, amino acid sequences of PvMSP-1_42_, PvAMA-1DII and PvDBPII were aligned with the homologous total (IgM + IgG) antibody responses assayed by ELISA against the recombinant protein of each antigen, and the seven (P01-P07) PvAMA-1DII peptides. Though Anti-PvMSP-1_19_ and anti-P04 antibody prevalence precludes strain-specific immune responses against the highly conserved PvMSP-1_19_ and the P04 peptide on domain II loop of PvAMA-1, a protective immune response was clearly elicited to these two regions by the marked isotype switch from IgM to IgG with increasing exposure in areas of endemicity. Further, the presence of strain transcending (cross reactive) PvDBPII specific functionally active binding inhibitory antibodies were demonstrated among locally exposed *P. vivax* patients. Thus this study may support the inclusion of PvMSP-1_19_, P04 region of PvAMA-1 domain II and region II of PvDBP as veritable vaccine components for a multi component "cocktail" asexual stage vaccine against *P. vivax* malaria in Sri Lanka.

